# Argentine tango: Another behavioral addiction?

**DOI:** 10.1556/JBA.2.2013.007

**Published:** 2013-06-14

**Authors:** Remi Targhetta, Bertrand Nalpas, Perney Pascal

**Affiliations:** ^1^Service d’Addictologie, CHU Caremeau, Nîmes, France; ^2^Inserm U1016, Nîmes, France

**Keywords:** addiction, tango, behavior, dependence

## Abstract

*Background:* Behavioral addiction is an emerging concept based on the resemblance between symptoms or feelings provided by drugs and those obtained with various behaviors such as gambling, etc. Following an observational study of a tango dancer exhibiting criteria of dependence on this dance, we performed a survey to assess whether this case was unique or frequently encountered in the tango dancing community. *Methods:* We designed an online survey based on both the DSM-IV and Goodman's criteria of dependence; we added questions relative to the positive and negative effects of tango dancing and a self-evaluation of the degree of addiction to tango. The questionnaire was sent via Internet to all the tango dancers subscribing to “ToutTango”, an electronic monthly journal. The prevalence of dependence was analyzed using DSM-IV, Goodman's criteria and self-rating scores separately. *Results:* 1,129 tango dancers answered the questionnaire. Dependence rates were 45.1, 6.9 and 35.9%, respectively, according to the DSM-IV, Goodman's criteria and self-rating scores. Physical symptoms of withdrawal were reported by 20% of the entire sample and one-third described a strong craving for dancing. Positive effects were high both in dependent and non-dependent groups and were markedly greater than negative effects. Long practice of tango dancing did not modify the dependence rate or reduce the level of positive effects. *Conclusions:* Tango dancing could lead to dependence as currently defined. However, this dependence is associated with marked and sustained positive effects whilst the negative are few. Identifying the precise substratum of this dependence needs further investigation.

## Introduction

For the World Health Organization, addiction is now considered to be a neurobiological disease (World Health Organization, 2004) defined as a compulsion to use a drug and the onset of withdrawal symptoms when consumption stops. At first restricted to drug and alcohol misuse, the concept of addiction has been subsequently extended to “pathological” behaviors such as exercise addiction (Allegre, Souville, Therme & Griffiths, 2006), compulsive buying, sexual hyperactivity (Duarte Garcia & Thibaut, 2010), computer/video playing and gambling, this list being still open for debate (Holden, 2010).

Tango is a popular dance for two, which originated in Rio de la Plata, Argentina, in the mid-19th century. Although several styles exist, tango is mostly danced in either open or close embrace, with long elegant steps and complex figures often with sensual connotation. Dancers, men and women, wearing specific clothes and shoes, are perfumed and very elegant.

The first author of this article (RT) is a physician specialized in addiction and an experienced tango dancer. At the end of a 10-day tango festival, he noticed a dancer presented by the tango teacher as the only dancer who attended the milonga (place for tango dancing) every night from the opening to the end of the session. RT developed a friendly relationship with this dancer and throughout their discussions RT suspected this dancer could be “addicted” to tango. Therefore, RT proposed to the dancer to conduct a complete interview, aiming to verify this hypothesis: He was a white collar in an insurance firm and has a very good income; he suddenly stopped working at 52 years of age in order to practice more and more tango as he wanted; then he moved to Argentina for 2 years to improve and intensify his practice; in Buenos Aires he danced every day from 11 PM to 4 AM and moreover spent 2 hours at least for preparation; he has never considered to reduce or stop dancing and, conversely, he started liking dancing more and more because he was feeling growing pleasure. He claimed that this practice presented no drawback, and on the contrary, there have been advantages such as well-being and self-confidence. Finally, the only time he did not dance was during a holiday week, he developed symptoms looking like those observed during withdrawal such as sadness, feeling uncomfortable and leg prickling. RT concluded that the relationship of this dancer with tango could correspond to an addiction which substantially differed from exercise dependence on sports such as running (Sachs & Pargman, 1979; Thornton & Scott, 1995) or body-building (Smith & Hale, 2005), since tango dancing requires usually smooth physical effort, it is always performed in an arousing senses environment, while embracing consecutively different partners. This unpublished observation became the supporting argument of the thesis that RT defended for his addiction certification in 2009.

Tango dancing has been reported to be an alternative therapy for balance, turning and moving improvement, particularly in patients with Parkinson's disease (Duncan & Earhart, 2012; Hackney & Earhart, 2009) and an efficient adjunct for the treatment of depression (Pinniger, Brown, Thorsteinsson & McKinley, 2012) but no study describing a possible addiction to tango has been published as stated by an extensive research in PubMed, Web of Science, using the keywords “tango, Argentine tango, addiction, dependence”.

Taking into account the case observed, the question was to assess whether tango dancing could lead to addictive behavior or was the dancer a highly specific and unique example. We therefore conducted a survey in the community of tango dancers in France.

## Methods

### Participants

The targeted sample was made up of all the subscribers to the journal “ToutTango”, an electronic and printed monthly newspaper devoted to tango dancing. At time of the study, about 15,000 persons were registered in the database of the journal. In the November 2011 issue, an advertisement was posted in the journal explaining that a survey aiming to evaluate whether tango dancing could be an addictive behavior was to be conducted the following month; this advertisement also explained the reason for such a study and its modalities; it requested all tango dancers to participate in the survey, even those only practicing occasionally or novices. The survey was entitled “Are you tango addicted?”

### Measures

Several screening questionnaires of addictive behaviors did exist, however, all are specific of a given behavior such as exercise (Exercise Dependence Scale [Downs, Hausenblas & Nigg, 2004]; Exercise Addiction Inventory [Terry, Szabo & Griffiths, 2004]), gambling (South Oaks Gambling Screen [Lesieur & Blume, 1987]); DSM IV-TR diagnostic criteria of pathological gambling (Stinchfield, Govoni & Frisch, 2005), body building (Bodybuilding Dependency Scale [Smith, Hale & Collins, 1998]), Internet (Internet Addiction Test [Young, 1998]; Compulsive Internet Use Scale [Meerkerk, Van Den Eijnden, Vermulst & Garretsen, 2009]) and so on, but none of them specifically fits with dancing. Moreover, as it was presumed that tango addiction did not fit well with exercise dependence, the corresponding diagnostic questionnaires did not appear to be appropriate for this study. As all these tools are derived from DSM-IV (American Psychiatric Association, 2000) criteria for substance dependence (tolerance, withdrawal, relapse, conflicts, etc.) we built a questionnaire based upon DSM-IV by re-writing each criterion to adapt them to tango, but without modifying their actual meaning; to complete our evaluation toolbox, we also adapted the Goodman's diagnostic criteria for addictive disorders (Goodman, 1990) (Table 1) and, secondly, we added a Likert scale from 0 to 5 for self-evaluation of the degree of addiction to tango. All items from DSM-IV and Goodman could be included except that corresponding to the “repeated efforts to reduce or stop” (E3 from Goodman's criteria and item 4 from DSM-IV) since this was not a concern for tango dancers, as stated by both the dancer's interview and RT experience. To fit with the future DSM-V definition of substance use disorders, we added a question regarding craving for tango (Q22). On the basis of the information recorded from the dancer's interview, we added some specific and hedonic questions related to the positive (physical or psychological) effects (Q6, Q8, Q17, Q24) and some items related to the negative (physical or psychological) effects (Q5, Q21, Q23, Q25) experienced. Answers were given on a Likert scale ranging from 0 (fully disagree or never, depending on the question) to 5 (completely agree or always, depending on the question).

**Table 1.** Tango dancing questionnaire according to DSM-IV and Goodman's criteria

Correspondenc to Goodman^1^ criteria	DSM-IV^2^	Effects	Questions
A			Q1. It's difficult for me not to dance
E1, E6	6		Q2. I organize my vacation in relation to tango dancing
E7	7		Q3. I dance even if I am injured or ill
	2b		Q4. After several days without dancing, I have to dance to feel good
E7	7	Negative	Q5. Tango dancing has persistent negative effects on my life (familial, social, professional or psychological)
		Positive	Q6. The more I dance, the more pleasure I have
E5	6		Q7. I go dancing although I have other things to do
		Positive	Q8. Tango dancing benefits my physical health (weight, cardio-vascular system, etc.)
	2b		Q9. I dance to avoid withdrawal symptoms
	4		Q10. I would like to dance more
E6	6		Q11. I have given up or reduced other important activities (familial, social, occupational, recreational) because of dancing
C			Q12. I feel reassured, my nervous tension goes down when I dance
E8	1a		Q13. At the beginning of tango dancing, I needed to increase my time of dancing (excepted that devoted to learning)
E7			Q14. A large part of my income is devoted to tango
B			Q15. I feel an increasing sense of tension immediately prior to dancing
D-E2	3		Q16. I dance longer than intended
		Positive	Q17. Tango dancing benefits my life (familial, social, professional or psychological)
E1			Q18. All the clothes, shoes, music that I buy are in relation with tango
E9	2a		Q19. I have withdrawal symptoms (feel bad, impatience, irritability, frustration, restlessness) if I cannot dance for several days
E1-E4	5		Q20. I spend a great deal of time for preparing and/or recovering (dressing, sleep, etc.)
		Negative	Q21. Has a relative or friend been concerned about your dancing?
			Q22. I have craving (irresistible urge) for dancing
E7	7	Negative	Q23. Tango dancing has negative effects on physical health (injury, articular pain, etc.)
		Positive	Q24. Thanks to tango, I feel better in my life (stress, self-confidence, self-esteem, etc.)
E7, D		Negative	Q25. I spend more money than expected on tango
E1	5		Q26. I think only about tango and listen only to tango music

^1^ Criteria numbering from Goodman (1990);

^2^ Criteria numbering from DSM-IV (American Psychiatric Association, 2000).

According to DSM guidelines, dependence was suspected when at least 3 criteria out of the 6 listed were positive. For Goodman's score of addictive disorder, we used his own specifications: dependence was suspected when a subject met at least A, B, C, D and five E criteria (Goodman, 1990) (Table 1), i.e. 9 criteria at least. When more than one question of our questionnaire corresponded to a given DSM or Goodman criterion (ex: Q2, Q7 and Q11 corresponded to DSM criteria Nr. 6), a positive answer to one of these questions was sufficient for criteria validation. A positive answer was defined as a score equal to or higher than 4 on the Likert scale. As a diagnostic criteria was omitted in both questionnaires (E3 from Goodman's criteria and item 4 from DSM-IV, see above), this could lead to underestimate but never overestimate the dependence rate.

At the end of the questionnaire we added socio-demographic items (age, sex), tango dancing items (frequency of dancing, time spent at the milonga, number of years of tango practice, being a tango teacher) and finally a Likert scale from 0 to 5 for self-evaluation of addiction to alcohol, tobacco, cannabis, psychostimulant and other addictive behavior (eating, sex, money, work, etc.).

### Procedure

The survey started on December 21, 2011 with a message on the Facebook page of the tango journal and then through a personal e-mail to each subscriber giving the web link to access to the questionnaire. A recall was sent on January 21, 2012 and the survey ended on January 31, 2012. The questionnaire was powered and hosted by a company dedicated to online surveys (www.sondageonline.com^®^). An integrated control checks IP addresses and does not authorize two answers from the same computer.

### Statistical analysis

Variables were described by means and standard deviations. Comparisons were made using either Anova with post-hoc tests when several sub-groups were compared or Student's *t*-test. Categorical variables were compared using *Chi^2^*-test or Fisher's exact test, when necessary. A *p* value of less than0.05 was considered as significant. All analyses were done using SPSS V15 software (SPSS Inc, Chicago, IL, USA).

## Results

### Description of participants

**Figure 1.**
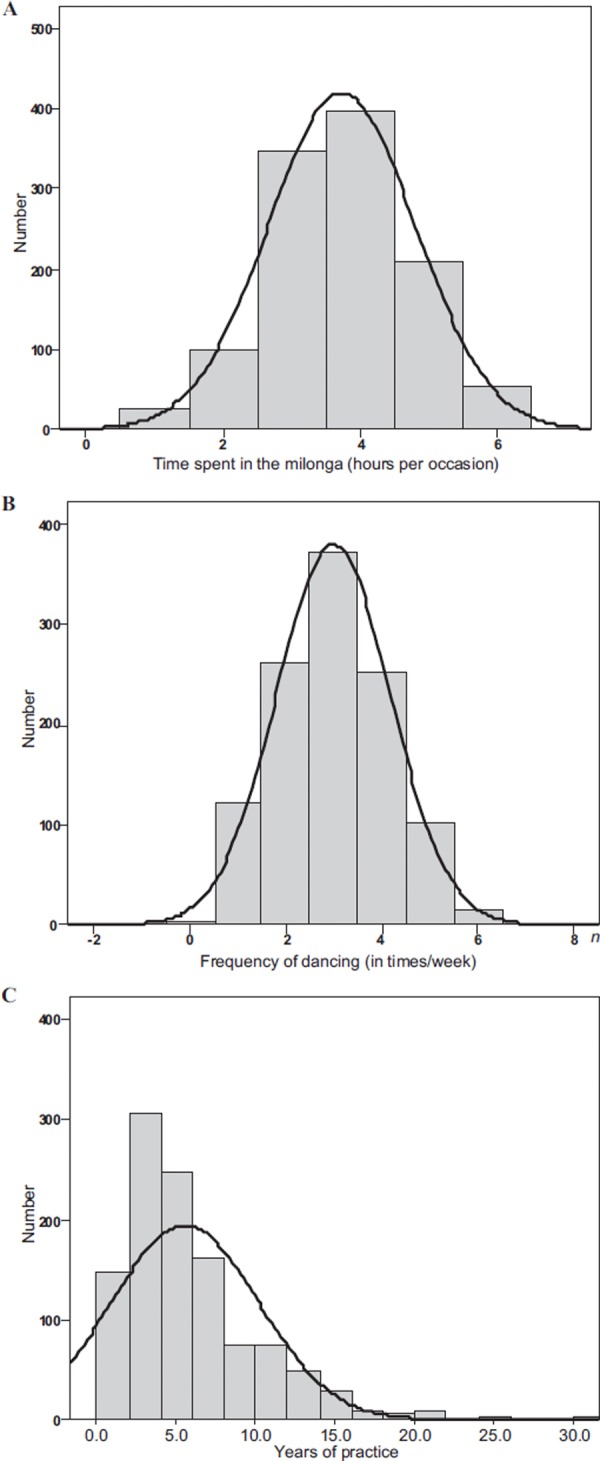
Distribution of values of time spent in the milonga (A), frequency of dancing (B) and years of tango practicing (C) among the whole sample

One thousand and three hundred seventy-two tango dancers participated in the survey, i.e. about 9.1% of the target sample, of whom 1,224 completed the questionnaire. Ninety-five were tango teachers and were excluded leading to keep 1,129 subjects in the analysis. The distribution of the time spent at the milonga and the frequency of dancing followed a Gaussian curve while that of years of practice was left-switched, demonstrating that more “beginners” than experienced dancers answered to survey (Fig. 1). There were 674 women (59.7%) and 455 men (40.3%) with a mean age of49.5 ± 13.1 years. Their main characteristics are presented in Table 2. The average practice of tango was longer for males than females (6.1 ± 5.2 vs. 5.1 ± 4.0, *p* < 0.001), 41.7% spent less than 3 hours and 23.2% more than 4 hours at the milonga per occasion, and half of them (55.3%) went dancing 2 to 3 times a week. Women spent significantly more time at the milonga per occasion but their frequency of dancing was significantly less.

**Table 2.** Characteristics of the sample studied

	All	Male	Female	*p*
*N*(%)	1129	455 (40.3)	674 (59.7)	
Age^1^	49.9 ± 13.1	52.8 ± 12.5	47.4±13.1	0.0001
Years of tango dancing^1^	5.5 ±4.6	6.1 ± 5.2	5.1 ±4.0	0.0001
Time spent in the milonga (%)^2^				
<2h	10.9	12.9	9.5	0.0005
2–3 h	30.8	32.1	29.9	
3–4 h	35.1	36.7	34.0	
≥4h	23.2	18.2	26.6	
Frequency of dancing (%)				
≤ once/week	34.3	29.5	37.5	0.001
2–3 times/week	55.3	57.6	53.7	
4 and more/week	10.5	13.0	8.8	
Self-rated addiction score^3^ to				
Tango	3.7±1.6	3.5 ± 1.6	3.7±1.6	0.02
Tobacco	1.6±1.4	1.5 ± 1.4	1.6±1.4	NS
Alcohol	1.4±0.8	1.5 ±0.9	1.3 ±0.8	0.002
Cannabis	1.0 ±0.4	1.0 ±0.4	1.0 ±0.3	NS
Stimulant	1.0 ±0.2	1.0 ± 0.1	1.0 ±0.2	NS
Behavior	1.8±1.2	2.0 ± 1.3	1.7±1.2	0.001

^1^ Mean ± *SD*; ^2^ per occasion; ^3^ maximum; score = 5; NS = not significant.

### Prevalence of dependence

The rate of dependence on tango varied according to the tool used for measuring (Table 3). Indeed, according to DSM-IV, Goodman's procedure and self-evaluation, 45.1%, 6.9% and 35.9% were dependent dancers, respectively. The score of self-rated addiction was significantly correlated both with DSM score (*r* = 0.67, *p* < 0.01) and the Goodman score (*r* = 0.69, *p* < 0.01); it increased significantly according to the number of DSM-IV positive criteria, from 3.3 ± 1.2 to 4.4 ± 0.8 in those having 3 and 6 positive criteria, respectively. Conversely, the self-rated addiction score did not change according to the number of Goodman's positive criteria. Finally, the mean self-rated addiction score was significantly higher in the group of dancers dependent on tango according to Goodman than in those qualified as dependent according to DSM-IV (4.3 ± 0.8 vs. 3.7 ± 1.1, *p* < 0.001).

**Table 3.** Rate of dependence on tango according to DSM-IV, Goodman's criteria and self-rating

Dependence according to	*N* positive criteria	*N* subject concerned	% dependent among total (n = 1129)	Self-rated score (Mean ± *SD*)
DSM-IV	3	174	15.41	3.31 ± 1.21^1^
	4	139	12.31	3.67 ± 1.10
	5	105	9.30	4.05 ±1.02
	6	92	8.15	4.42 ± 0.84
	Total	510	45.17	3.76 ± 1.15^2^
Goodman	9	15	1.33	4.20±0.86^3^
	10	14	1.24	4.36 ±0.93
	11	30	2.66	4.33 ±1.03
	12	19	1.68	4.52 ± 0.61
	Total	78	6.91	4.36 ± 0.88
Self-rated score	4	236	20.90	
	5	170	15.06	
	Total	406	35.96	

^1^ All the self-rating scores were significantly different (*p* < 0.03 at least) from each other.

^2^ *p* < 0.001 vs. self-rating score in Goodman dependence group.

^3^ There was no significant difference between self-rating scores.

### Comparison between the three diagnostic methods

**Figure 2.**
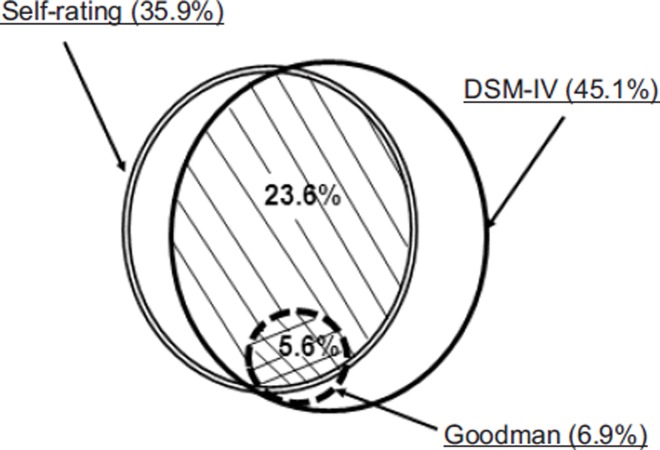
Prevalence of tango dependence according to DSM-IV, Goodman's criteria and self-rating and their combination in the whole sample (*n* = 1129). Dash line circle: dependence according to DSM-IV (*n* = 510, 45.1%). Double line circle: dependence according to self-rating (*n* = 406, 35.9%); dotted line circle: dependence according to Goodman's criteria (*n* = 78, 6.9%); simple grid: subjects dependent with both DSM-IV and self-rating (*n* = 266,23.6%); double grid: subjects dependent with DSM-IV and self-rating and Goodman's criteria (*n* = 64, 5.6%)

Cross comparisons between the three methods of dependence screening showed that about two-thirds (64.7%) of the DSM-IV dependent dancers qualified themselves as dependent through the self-rating scale, while only 15.3% were identified as so using Goodman's criteria (Table 4). Among those self-identified as dependent, 81.3% and 15.8% fulfilled to DSM-IV and Goodman's criteria, respectively. Finally Goodman's classification showed the best concordance rate with the two other modalities, 100% with DSM-IV and 82.1% with self-rating, however, this classification provided a very low prevalence of dependence on tango in the whole sample. When dependence was defined by a positive score in two methods (DSM-IV + self-rating), the prevalence was 23.6% and fell to 5.6% when combined with a positive score for Goodman's criteria (Fig. 2). The combination of self-rating plus Goodman's criteria led to a5.6% prevalence and was independent of DSM-IV since, as stated above, all the subjects positive to Goodman's criteria were also positive to DSM-IV.

**Table 4.** Cross-validation of dependence on tango between DSM-IV, Goodman's criteria and self-rating

	*N* dependent (%)	Dependence (%) according to		
		DSM-IV	Goodman	Self-rating
DSM-IV	510(45.1)		15.3	64.7
Goodman	78 (6.9)	100		82.1
Self-rating	406(35.9)	81.3	15.8	

All the items of the Goodman and DSM-IV dependence score increased with the self-rated score of addiction, however, the 3 items relative to physical withdrawal symptoms (Q4, Q9, Q19) were those showing the highest difference between non- and severely-addicted dancers. A detailed analysis of these items showed that 17.4% of all the responders scored 4 or 5 to item 19, a proportion which rose to38.7% when only those who estimated themselves to be severely tango addicted (*n* = 406) were considered. Among the 510 dancers showed to be dependent by DSM-IV, 92.9% met the criteria of physical dependence.

### Craving

Craving for dancing was a highly frequent feeling in our sample. Indeed the median value of this item was 3, and33.7% of the sample scored 4 or 5; conversely only 8.8% scored 0. As expected, the craving score increased significantly (*p* < 0.001) according to self-rated addiction from 1.3 ± 1.2 in those scoring 0 to 4.1 ± 1.0 in those scoring 5; a similar significant increase was found when dependence was assessed using DSM-IV (1.9 ± 1.3 vs. 3.5 ± 1.2) or Goodman's criteria (2.6 ± 1.4 vs. 4.2 ± 0.9). Altogether, 58.6% and79.5% of those dependent on tango according to DSM-IV and Goodman's criteria had a high score for craving (= 4), respectively.

### Consequences

**Figure 3.**
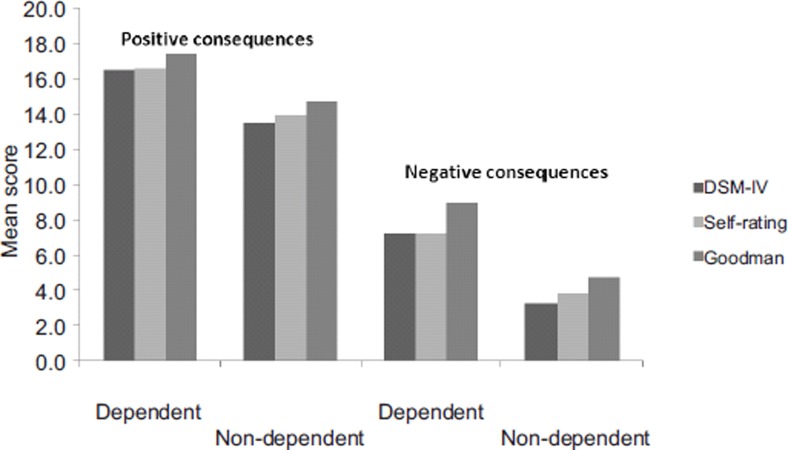
Positive and negative effects according to dependence on tango evaluated using DSM-IV, Goodman's criteria and self-rating. Positive/negative effects were the sum of the 4 item scores concerned, respectively. Each item score ranged from 0 to 5

The questionnaire included four items dealing with the negative effects and four items dealing with the positive effects of tango dancing on health and personal behavior, the maximum score being 20. The score of the positive effects was spontaneously high in non-addicted dancers and increased significantly in those identified as dependent (Fig. 3); results were similar whatever the dependence classification used. Conversely the score of negative effects was very low in non-addicted dancers; it increased significantly in addicted dancers (Fig. 3) but the score for positive effects was always twice as high as that of negative effects.

**Figure 4.**
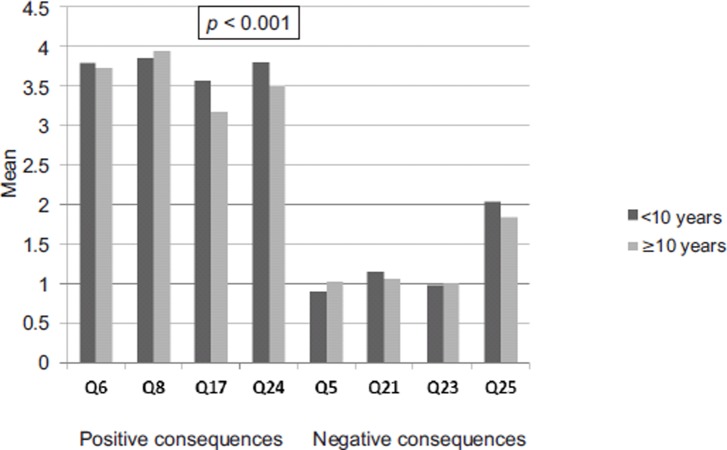
Comparison of positive and negative effects according to the degree of tango practice (< 10 years, *n* = 942; ≤ 10 years, *n* = 187). Q17 and Q24 scores were significantly lower in dancers with more than 10 years of practice. (Q6 = pleasure; Q8 = physical health; Q17 = benefits to my life; Q24 = feel better; Q5 = negative to my life; Q21 = relative; Q23 = physical health; Q25 = money)

One hundred and eighty-seven (16.6%) dancers had 10 and more years of tango practice. They were significantly older than those who had started dancing more recently(57.2 ± 8.6 vs. 48.0 ± 13.3 years, *p* < 0.001). The prevalence of dependence did not differ from that calculated for subjects who had less practice whether estimated through DSM-IV (41.2 vs. 46.1%, *p* = 0.26), Goodman's criteria (6.4 vs. 7.0%, *p* = 0.75) or self-rated (3.6 ± 1.6 vs. 3.7 ± 1.6, *p* = 0.82). Experienced dancers were slightly, although significantly, less prone to dance when ill or injured; they also had slightly less positive effects (14.3 ± 4.0 vs. 15.0 ± 3.9, *p* = 0.03) but their pleasure while dancing (Q6) was still high and did not differ from that of less experimented dancers(3.8 ± 1.3 vs. 3.7 ± 1.2, *p* = 0.54); finally, negative effects did not increase with time and were similar in the two groups(4.9 ± 4.0 *vs.* 5.0 ± 4.0, *p = 0.83*) (Fig. 4).

### Characteristics of dependent dancers

For each dependence classification (DSM-IV, Goodman's criteria, self-rating), we compared the characteristics of dependent to those of non-dependent dancers (Table 5). As the results found with each classification were not statistically different, only those obtained using DSM-IV are shown: 1) the rate of dependence did not differ according to sex; 2) dependent dancers were one year younger than non-dependent dancers but the difference was not significant; however, when age was adjusted by sex, dependent women were significantly younger than men; 3) the number of years of tango dancing did not differ between groups; 4) the mean self-rated scores for tobacco, alcohol, cannabis and psychostimulant addiction were rather low in both dependent and in non-dependent dancers; dependent tango dancers had a slight, although significant, increase in the mean score of tobacco addiction; 5) dependent dancers went to the milonga more frequently; 6) 75.7% of dependent dancers spent 3 hours or more at the milonga on one occasion against24.3% of non-dependent dancers (*p* < 0.001); 7) about half (48.1%) of the dependent dancers spent more than one hour per day for tango preparation against 18.3% (*p* < 0.001) of the non-dependent dancers.

**Table 5.** Comparison between dependent and non-dependent dancers classified according to DSM-IV

	Non-dependent	Dependent
Sex M (%)	56.5	43.5
W (%)	53.7	46.3
Age M	53.3 ± 12.4	52.1 ± 12.6^1^
W	47.7 ± 12.7	47.1 ± 13.6
Years of tango practice	5.7 ± 5.0	5.4 ±4.1
Frequency of dancing		
≥ 3 times/week (%)	18.9	49.6^2^
Time spent in the milonga		
≥ 3 hours per occasion (%)	46.4	73.2^2^
Time devoted to tango preparation		
≥ 1 hour per day (%)	15.2	40.4^2^
Craving^*^	1.9±1.3	3 .6 ± 1.2^1^
Self-rate of addiction to^*^		
Tobacco	1.5 ± 1.3	1.7 ± 1.5^2^
Alcohol	1.4±0.8	1.5 ± 0.9
Cannabis	1.0 ±0.3	1.1 ± 0.4
Psychostimulant	1.0 ± 0.1	1.0 ±0.2
Behavior	1.7±1.1	1.9 ± 1.3^2^

^*^ Maximum score = 5.

^1^ *p* < 0.001 vs. dependent men.

^2^ *p* < 0.05 vs. non-dependent.

## Discussion

Individuals may have a passion for one or more specific behaviors and repeat them in order to experience the corresponding reward and/or to satisfy its desire. However, in some cases the behavior is repeated while personal or environmental conditions are not adequate, thus leading to negative effects. Incapacity to refrain from such behaviors is a specific trait of dependence, leading to the concept of behavior addiction (Grant, Potenza, Weinstein & Gorelick, 2010) in parallel to substance addiction.

Issues relative to behavioral addictions are currently being debated in the context of the development of the fifth edition of the DSM. Currently, only one behavioral addiction, pathological gambling, is a recognized diagnosis in DSM-IV and ICD-10, with criteria conceptually similar to those for substance abuse/dependence. In our study, we have adapted the criteria of DSM-IV and Goodman to Argentine tango and our analytical procedure followed the method currently in use.

According to our results, tango dancing satisfies several criteria of addiction: feelings of tension or arousal and craving state before dancing, pleasure or relief when dancing, tolerance characterized by a need to increase time spent dancing, and finally physical withdrawal symptoms following abstinence. Altogether this suggests that dependence on tango could exist.

Dependence on tango led to negative effects; however, their scores remained rather low even if they were doubled in dependent dancers. On the other hand, dependence on tango was characterized by positive and specific effects; scores which were already high in non-dependent dancers increased further with the development of dependence. Moreover, positive effects were always twice as high as negative effects both in dependent and non-dependent dancers.

Non-dependent and dependent dancers had similar and very low self-rated scores for addiction to alcohol, cannabis and other behavioral addictions; only the addiction score to tobacco was slightly, but significantly, higher in dependent dancers. These results, which are consistent with observational study (RT, personal communication), might suggest that tango dancers are not specifically at risk of any addiction but this needs to be confirmed by studies based on validated diagnostic tools. In other behavioral addictions, the prevalence of co-dependence seems to vary according to the behavior: high prevalence of anorexia in professional ballet dancers (Pierce, Daleng & Mcgowan, 1993), frequent substance use disorders in pathological gamblers or compulsive sexual addicts (Grant, 2008), lower rate of smokers in exercise dependent subjects (Lejoyeux, Avril, Richoux, Embouazza & Nivoli, 2008).

Tango dancing is known to have a therapeutic effect. Indeed tango was shown to improve activity in patients with Parkinson's disease (Foster, Golden, Duncan & Earhart, 2013). In elderly seniors at risk of falling, tango seemed to result in greater improvement in balance skills and walking speed than did walking (McKinley et al., 2008), and can be useful as rehabilitation in patients with chronic stroke (Hackney, Hall, Echt & Wolf, 2012). These results are confirmed by our survey which showed that almost all responders claimed that tango dancing led to positive physical effects which are maintained throughout their practice.

The self-rated score was closely correlated to that of the two other tools but, conversely to a recent study targeted on Internet addiction but using a similar design than the present one (Widyanto, Griffiths & Brunsden, 2011), the three tools used to diagnosis dependence gave very different results with prevalence ranging for 6.9% (Goodman) to 45.1% (DSM-IV); the self-rating tool resulted in a prevalence(35.9%) that was closer to that obtained with DSM-IV than with Goodman's criteria. The difference in prevalence may come from an inadequate conceptualization of the phenomenon to identify or from differences in the screening tools used (Hausenblas & Symons Downs, 2002). Indeed, in DSM-IV the existence of dependence being suspected as soon as 3 criteria (out of 7), whatever they are, are positive. In Goodman's questionnaire, as compared to DSM-IV, more stringent conditions are requested for suspecting an addiction: the number of positive criteria is higher (at least 9 out of 12) and 4 specific criteria (A, B, C, D) should mandatorily be positive. Such a heterogeneity can explained at least in part the discrepancies in our prevalence results as it has already been found in exercise addiction (Berczik et al., 2012). The detailed analysis of our DSM-IV score results showed that about one-third of those identified as dependent met 3 criteria only. Increasing the DSM-IV cut-off for dependence to 4 criteria would substantially reduce prevalence in our sample; to achieve the prevalence rate obtained through Goodman's criteria with DSM-IV we would need to raise the cut-off to at least 6 criteria (see Table 2). Hence, the number of criteria could constitute the severity specifiers of tango addiction, from moderate to severe, as proposed in the future DSM-V. It should be noted that all subjects meeting Goodman's dependence criteria were also dependent according to the DSM-IV whilst the opposite was not true. Most dancers self-rating themselves as dependent also met Goodman's criteria. Altogether, only 64 dancers (5.6%) were dependent according to all three screening methods; they could well represent the hard core of dependent dancers.

About 10% of the potential responders participated in the survey and we had no means to evaluate whether our sample was representative of the tango dancing community. Only a few details are available on this topic; in a recent survey, Kreutz (2008) described tango dancers as “highly educated individuals strongly involved in tango dance as a primary leisure activity” but his sample was non-randomized and made up of 110 subjects only. More than 1,100 dancers participated in our survey but one can suspect that those who felt most concerned by the topic participated more than others. However, all dancers were clearly asked to participate, either beginner or experimented, either occasionally or frequently practicing. Such objective seemed to have been reached since, after exclusion of tango teachers (*n* = 95, see results), the answers to questions on time spent in the milonga, frequency of dancing followed a Gaussian distribution while that of years of practice was left-switched towards beginners, however, a selection bias cannot definitely be excluded.

Altogether, our results strongly suggest that tango dancing might lead to dependence as defined in the current diagnosis manual. Why some dancers become dependent on tango and what is the precise substratum of this dependence, i.e. primary or secondary, remains unknown as yet; however, the low score in self-evaluation of other addictive behavior (eating, sex, money, work) observed in our sample favors a primary addiction and not a secondary one as reported in exercise dependence (Berczik et al., 2012). Tango dependence is associated with several strong and sustained positive effects (pleasure, self-esteem, reduced stress, physical health, etc.) while negative effects are weak. This looks like what has been described for exercise addiction (Berczik et al., 2012). Indeed, as tango, as well as all dances, includes physical activity, one can wonder whether tango dependence could be a simple variant of exercise addiction. However tango cannot be summed-up to a simple exercise. Indeed this dance includes several environmental specificities such as dressing up, music, social status and perfume, sensuality provided by the close embrace of partners during dancing (abrazo) and the possibility of embracing several different partners according to the usual tenda (succession of 3 or 4 tangos) protocol, all these sensations leading to “tango drunkenness”. Owing to the latter reasons, it seems likely that tango addiction resembles only partly to exercise addiction. Nevertheless further studies are needed in this regard once the concept of behavioral and exercise addiction will be definitely conceptualized (Berczik et al., 2012; Freimuth, Moniz & Kim, 2011).
